# Animal listeriosis in Germany: An update for the current situation over 2 years (2024–2025) and the need for a surveillance system in the animal health sector

**DOI:** 10.1186/s13567-026-01755-5

**Published:** 2026-05-09

**Authors:** Gamal Wareth, Sven Halbedel, Heinrich Neubauer

**Affiliations:** 1 Institute of Bacterial Infections and Zoonoses, Friedrich-Loeffler-Institut, Naumburger Str. 96a, 07743 Jena, Germany; 2 Institute of Infectious Diseases and Infection Control, Jena University Hospital, Jena, Germany; 3 FG11 Division of Enteropathogenic Bacteria and Legionella, Robert Koch Institute, Wernigerode, Germany; 4 Institute for Medical Microbiology and Hospital Hygiene, Otto Von Guericke University Magdeburg, Magdeburg, Germany

**Keywords:** Animal listeriosis, distribution, prevalence, TSN, Germany

## Abstract

Animal listeriosis is a sporadic bacterial infection caused by *Listeria* (*L*.) *monocytogenes* and *L. ivanovii.* In Germany, only listeriosis caused by *L. monocytogenes* is considered a notifiable disease. The objective of this report is to analyze official surveillance data on animal listeriosis in Germany from 2024 to 2025 to assess the epidemiological situation and the spatiotemporal distribution of this One Health relevant disease. A total of 341 notifications involving 652 affected animals were reported. The highest number of cases occurred in food-producing animals, particularly cattle, followed by sheep and goats. Listeriosis has also been reported in pet animals, wildlife, and poultry. The epidemiological situation and geographical distribution of the disease have remained consistent over the past decade, with the highest incidences in Berlin, Bavaria, and Baden-Wuerttemberg. Listeriae cause disease in a broad range of hosts nationwide. Monitoring listeriosis in animals is crucial for public health and the safety of the food supply. Systematic collection of animal isolates is essential to understand transmission from environmental reservoirs to humans—a currently puzzling link. This knowledge is vital for protecting human health.

## Introduction, methods and results

Listeriosis is a major zoonotic disease affecting both humans and various animal species worldwide. It is caused by *Listeria* (*L.*) *monocytogenes* and *L. ivanovii,* which share several common virulence factors [1]. This bacterium can multiply across a wide temperature range, from 4 to 44 °C, but is more common in colder environments. In animals, listeriosis typically results in septicemia, abortion, or latent infection [2]. In adult ruminants, the main clinical signs are encephalitis or meningoencephalitis and abortion [3]. A high prevalence of intestinal carriers with gastrointestinal issues is reported. Sepsis and mastitis, however, are uncommon [4]. Listeriosis can present with various clinical manifestations in sheep and goats [5]. The disease usually progresses more rapidly in goats than in sheep. The most common form causes inflammation of the brain and meninges, leading to a condition called “circling disease”. Abortion, sepsis, and involvement of the eye can also occur [6]. Listeriosis can affect wildlife, including foxes, raccoons, fallow deer, roe deer, wild boars, and poultry such as chickens, pigeons, and waterfowl [7–9]. Listeriosis occasionally occurs in wildlife species. When it does occur, it is generally associated with the consumption of spoiled feed. The clinical signs are similar to those observed in domestic ruminants and mainly include hepatitis, splenitis, pneumonia, and meningitis [8]. Overall, however, the disease is quite rare.

In Germany, listeriosis has been extensively studied in the food supply chain, and nearly all human outbreaks have been linked to the consumption of food of animal origin [10–12]. Despite this fact, animal listeriosis has received little attention [7]. Continuous monitoring of listeriae prevalence in livestock and subsequent reduction of germ load in primary production will ultimately result in a lower risk of product contamination and improved animal welfare. Analysis of notification data for listeriosis according to the EU Animal Health Law for Germany provides detailed information for the veterinary medicine sector to be linked with data on hygiene and human medicine, then. This analysis for 2024 and 2025 covers the spatial and temporal distribution of cases and the affected hosts across Germany's federal states.

**Figure 1:**
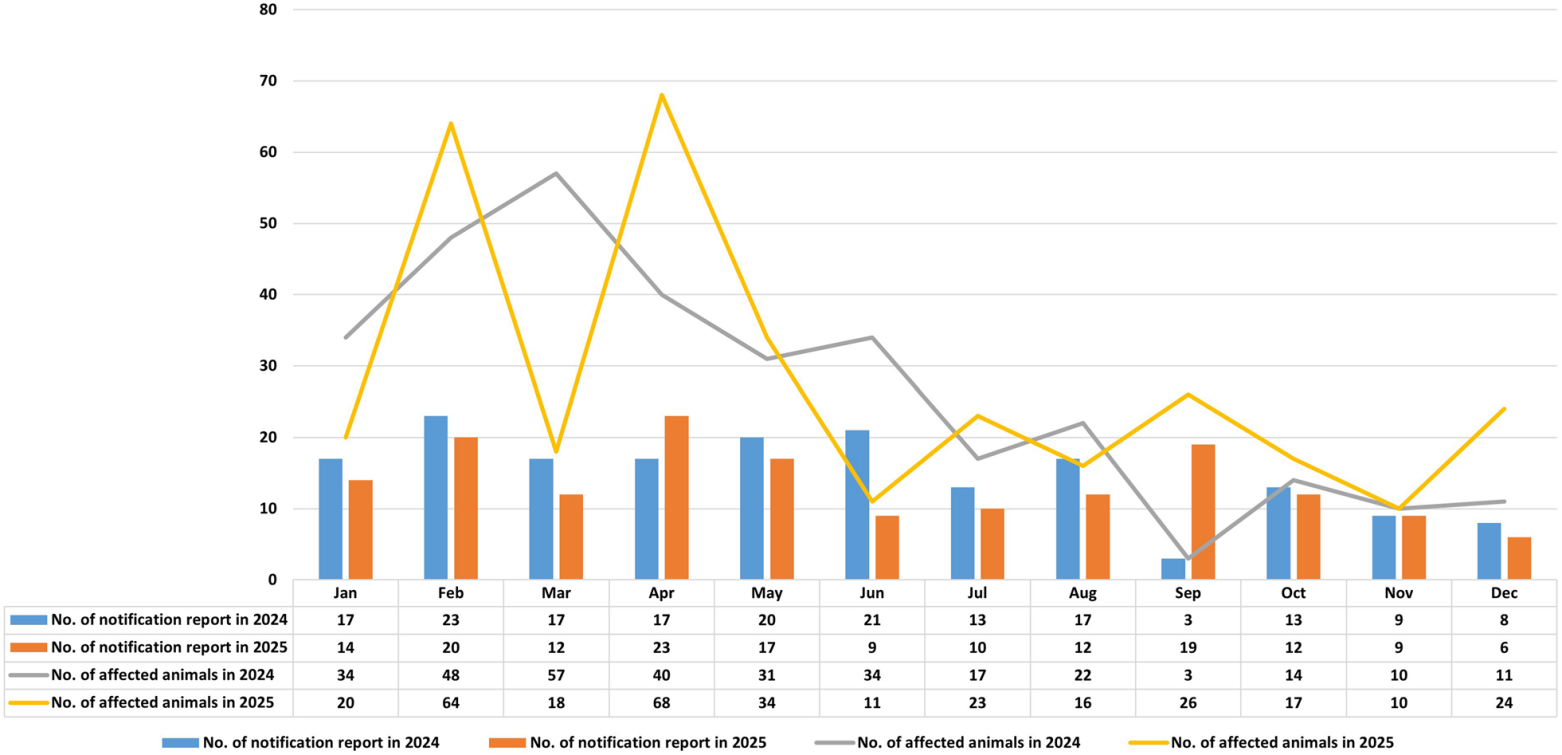
**Number of monthly registered notification reports and affected animals with listeriosis in Germany for 2024 and 2025.**

In this study, notification reports of outbreaks and cases of animal listeriosis caused by *L. monocytogenes* over two years (2024–2025) in Germany were retrieved from the Animal Disease Notification System (TierSeuchenNachrichten-System, TSN) and analyzed. The TSN is the platform for collecting data and information on cases and outbreaks of notifiable and reportable animal diseases occurring in Germany. Cases involving livestock, pets, and wildlife may be reported. The system aims to ensure compliance with national and international reporting obligations. Although both *L. monocytogenes* and *L. ivanovii* are considered pathogenic for animals, only listeriosis caused by *L. monocytogenes* is included in the mandatory notification reports. In total, 341 notification reports (NRs) were recorded over the two-years period from January 2024 through December 2025, involving 652 animals (Table 1, Figure 1). In 2024, 178 mandatory reports were registered for 321 animals, with 133 diseased and 155 dead. The month with the most affected cases was March (*n* = 57), followed by February (*n* = 48) and April (*n* = 40). In 2025, 163 NRs were recorded, affecting 331 animals, with 134 diseased and 175 dead animals. The peak months were April (*n* = 23 NRs, 68 affected animals) and February (*n* = 20 NRs, 64 affected animals) (Figure 1).

**Table 1:** **The number of mandatory notification reports for animal listeriosis based on the Animal Disease Notification System (TSN) reports queried**

Animal species	2024	2025	Total
Cattle	44	41	85
Sheep	49	35	84
Foxes	22	40	62
Goats	16	18	34
Poultry	7	8	15
Rabbits	5	3	8
Dogs	1	1	2
Equine	1	-	1
Swines	-	1	1
Others*	33	16	49
Total	178	154	341

Please note that multiple animal species can be associated within a single TSN report.

*Others include not identified animal species and other ungulates.

**Figure 2:**
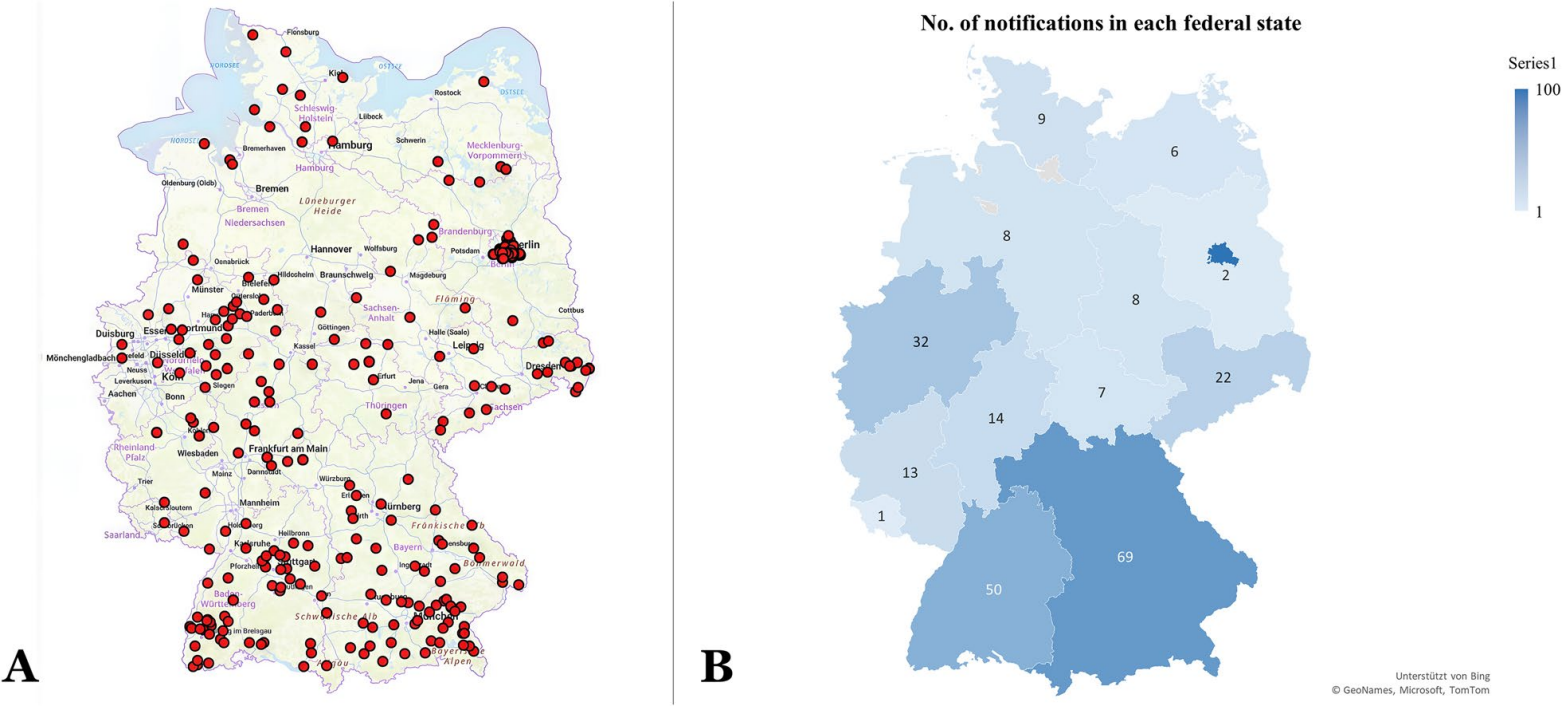
**Map showing the nationwide distribution of reported cases and outbreaks of animal listeriosis (A) and the number of notification reports (B) in each federal state of Germany for 2024–2025.**

Listeriosis was reported in nearly all species of livestock, as well as in companion animals and wildlife. The highest number of cases was reported in cattle (*n* = 85), followed by sheep (*n* = 84), foxes (*n* = 62), and goats (*n* = 34). Only two NRs were registered for dogs, and one each for a horse and a pig (Table 1). The geographical distribution of listeriosis was nationwide (Figure 2). Of the 16 federal states, 14 were affected. No cases were notified in Hamburg and Bremen, which have indeed only limited animal husbandry (Table 2). This trend was also observed over the last 10 years [7]. Berlin, Bavaria, and Baden-Wuerttemberg showed the highest numbers of cases, while Saarland and Brandenburg had the lowest (Figure 2, Table 2). The epidemiological situation of animal listeriosis and the spatial distribution of mandatory notifications over the last 2 years are consistent with, and almost identical to, the findings over the last decade as shown in Table 3 [7].

**Table 2:** **Spatiotemporal distribution of reported cases and outbreaks of animal listeriosis in Germany (2024–2025), based on the annual number of notifications in each federal state**

Federal states	2024	2025	Total
Berlin	51	49	100
Bavaria	37	32	69
Baden-Wuerttemberg	24	26	50
North Rhine-Westphalia	19	13	32
Saxony	7	15	22
Hessia	10	4	14
Rhineland-Palatinate	6	7	13
Schleswig–Holstein	6	3	9
Saxony-Anhalt	5	3	8
Lower Saxony	5	3	8
Thuringia	4	3	7
Mecklenburg-Pomerania	3	3	6
Brandenburg	–	2	2
Saarland	1	–	1
Total	178	163	341

**Table 3:** **Spatiotemporal distribution of reported cases and outbreaks of animal listeriosis in Germany (2014–2023), in each federal state, modified from Wareth and Neubauer **[7]

Year	2014	2015	2016	2017	2018	2019	2020	2021	2022	2023	Total
Bavaria	32	60	67	47	44	37	32	41	29	20	409
Berlin	8	10	8	40	21	12	31	30	26	35	221
Baden-Wuerttemberg	15	22	38	23	14	16	24	20	11	17	200
North Rhine-Westphalia	13	25	23	16	14	16	15	28	18	21	189
Saxony	18	23	31	22	17	11	14	13	11	16	176
Schleswig–Holstein	20	8	14	9	12	5	15	3	4	7	97
Hessia	8	17	9	9	3	11	8	7	10	8	90
Rhineland-Palatinate	5	8	2	9	9	3	3	5	7	5	56
Lower Saxony	9	10	9	5	6	4	4		4	2	53
Saxony-Anhalt	6	11	6	3	4	3	1	2	1	5	42
Brandenburg	4	11	1	7	2	2	4	2	2	2	37
Mecklenburg-Pomerania	1	4	6	4	5	5	2	1	–	1	29
Thuringia	2	6	4	3	2	3	–	2	3	2	27
Saarland	–	–	–	–	–	–	1	1	–	1	3
Total	141	215	218	197	153	128	154	155	126	142	1.629

## Discussion

Listeriosis is a severe foodborne illness in humans caused by the bacterium *L. monocytogenes.* It poses a significant threat, particularly to immunocompromised individuals [13]. This resilient, Gram-positive bacterium can replicate intracellularly, leading to severe disease. Outbreaks of human listeriosis have been reported worldwide, including Germany, and are mainly linked to the consumption of contaminated food of animal origin [14–17]. *Listeria monocytogenes* can survive in harsh environments, such as soil, decaying vegetation, and animals’ intestines. It is also found in various foods such as unpasteurized dairy products, soft cheeses, raw meat fish, seafood, vegetables, and fruits [18]. Compared to other bacterial foodborne infections such as salmonellosis, human listeriosis is rare, with an incidence of 0.2 to 0.9 cases per 100 000 inhabitants in high-income countries [12, 19]. However, the fatality rate remains notably high. Increasing awareness and implementing preventive strategies are critical to safeguarding vulnerable populations from this pathogen.

In animals, the disease is mainly caused by *L. monocytogenes*, while the role of *L. ivanovii* in animal disease remains unclear, as its pathogenic potential and contribution are not well defined. All data in the TNS notification reports refer exclusively to cases caused by *L. monocytogenes*, overlooking that *L. ivanovii* can also cause severe infections and shares pathogenic mechanisms and virulence factors with *L. monocytogenes* [20, 21]. Therefore, monitoring of *L. ivanovii* on animal farms, in food sources, and in the environment is needed to assess the risk to consumers and animals. Current notifications indicate that animal listeriosis affects a wide range of hosts in Germany and causes numerous outbreaks each year. Despite this, little attention is given to animal disease, and only limited information is available on *L. monocytogenes* in animals and the environment [22–25]. Thus, the impact of listeriosis on farm animal health and welfare remains to be determined. Notification of listeriosis in Germany relies on the accurate diagnosis and isolation of the causative bacterium, *L. monocytogenes*, at the state veterinary laboratories. To ensure precise diagnosis, samples are collected from diseased or deceased animals after clinical signs of listeriosis appear. Diagnosis is based on isolating the bacterium from relevant organs such as the brain, aborted materials in cases of abortion, or fecal samples in cases of digestive involvement, followed by identification using culturing and MALDI-TOF. Serotyping is not required; therefore, notification reports do not include serotype information. Consequently, serogrouping, susceptibility testing, and whole-genome sequencing of *Listeria* isolates from animals and their environments are essential to better understand the epidemiological situation and to identify drivers of resistance.

Following ingestion by farm animals, the bacterium can colonize or infect the host depending on the infectious dose. High-dose exposure results in diseases such as gastroenteritis, abortion, or neurological disorders, and the pathogen is subsequently excreted into the environment through feces [26]. Contamination of both animal- and plant-based foods may occur via infected animals or fecal contamination of crops. Asymptomatic colonization of ruminants and pigs with *L. monocytogenes* is common, particularly after low-dose exposure, with the pathogen detectable in feces and lymphoid tissues [24, 27]. This colonization facilitates the transmission of *L. monocytogenes* to bovine milk, carcasses, and unprocessed meat, which can ultimately result in listeriosis in humans.

Animal listeriosis is reported throughout Germany, but the number of notifications varies among federal states. Human listeriosis data from the Robert Koch Institute show that the highest incidence occurs in Thuringia, whereas the largest total numbers of cases are reported in North Rhine-Westphalia, Bavaria, and Baden-Wuerttemberg [16]. These three states, which also have the largest populations, along with Berlin, report the highest numbers of animal listeriosis cases as well. The parallel pattern seen in animal notifications and human cases, particularly in these populous states and Berlin, points to a potential link between animal and human listeriosis dynamics, suggesting shared risk factors, such as livestock density, farm practices, or local food contamination.

Surveillance of listeriosis in Germany currently relies on whole genome sequencing of *L. monocytogenes* isolates from infected patients, food, and food production environments [16, 28]. This has led to the detection of several, in some cases large, outbreaks of human listeriosis and to their termination by identifying the causative food vehicle. Systematic collection of *L. monocytogenes* isolates from animals and from the farm environment will complement this successful procedure and will enable tracing human listeriosis outbreaks back to food producing livestock or their feed and environmental reservoirs. The epidemiological situation and clinical presentation of animal listeriosis are similar in neighbouring and European countries, such as France [29], the Netherlands [30], Italy [31], Greece [32] and Türkiye [5]. However, the disease in farm animals at the primary production level often receives little attention and should be given more focus. The on-farm implementation of a systematic, nationwide collection of *L. monocytogenes* isolates from diseased farm animals, combined with whole genome sequencing and comparison with sequences from clinical and food isolates will help to reducing the incidence of listeriosis in both animals and humans finally.

## Data Availability

No datasets were generated or analysed during the current study.
